# Declining methane emissions and steady, high leakage rates observed over multiple years in a western US oil/gas production basin

**DOI:** 10.1038/s41598-021-01721-5

**Published:** 2021-11-16

**Authors:** John C. Lin, Ryan Bares, Benjamin Fasoli, Maria Garcia, Erik Crosman, Seth Lyman

**Affiliations:** 1 Department of Atmospheric Sciences, University of Utah, Salt Lake City, USA; 2 Department of Life, Earth and Environmental Sciences, West Texas A&M University, Canyon, USA; 3 Bingham Research Center, Utah State University, Salt Lake City, USA; 4 Present Address: Division of Air Quality, Utah Department of Environmental Quality, Salt Lake City, USA; 5 Division of Air Quality, Utah Department of Environmental Quality, Salt Lake City, USA

**Keywords:** Atmospheric science, Climate change

## Abstract

Methane, a potent greenhouse gas, is the main component of natural gas. Previous research has identified considerable methane emissions associated with oil and gas production, but estimates of emission trends have been inconsistent, in part due to limited in-situ methane observations spanning multiple years in oil/gas production regions. Here we present a unique analysis of one of the longest-running datasets of in-situ methane observations from an oil/gas production region in Utah’s Uinta Basin. The observations indicate Uinta methane emissions approximately halved between 2015 and 2020, along with declining gas production. As a percentage of gas production, however, emissions remained steady over the same years, at ~ 6–8%, among the highest in the U.S. Addressing methane leaks and recovering more of the economically valuable natural gas is critical, as the U.S. seeks to address climate change through aggressive greenhouse emission reductions.

## Introduction

Methane (CH_4_) is a key greenhouse gas whose concentrations have risen since the Industrial Revolution from a combination of natural and anthropogenic activities^1,2^. CH_4_ is the main constituent of natural gas which, when combusted, produces less carbon dioxide (CO_2_) per unit of energy compared to coal or petroleum^3^. However, if CH_4_ is leaked to the atmosphere from energy infrastructure without undergoing combustion, the climate benefits are outweighed^3,4^ by the high global warming potential of CH_4_–84 times that of CO_2_ over a 20 year time horizon^2^. As such, reducing CH_4_ emissions is an important means for climate change mitigation^5,6^, particularly over the next few decades^7^. Moreover, since CH_4_ is a precursor of tropospheric ozone (O_3_), a criteria pollutant regulated by the U.S. Environmental Protection Agency, air quality and health co-benefits can be realized from CH_4_ reductions in addition to mitigating climate change^8,9^.

CH_4_ emissions from oil and gas infrastructure in the U.S. have attracted growing scientific and policy attention as horizontal drilling and hydraulic fracturing significantly boosted U.S. production of oil and natural gas since the early 2000s, revolutionizing the nation’s energy production capabilities^10^. The past decade’s worth of research have revealed that: (a) Emissions can be dominated by a small number of sources (“super-emitters”), often associated with abnormal conditions or malfunctioning equipment^11–13^; and (b) inventories underestimate CH_4_ emissions, primarily in the production sector of the oil/natural gas supply chain^14^. Estimates point to emissions from the U.S. oil and natural gas supply chain to be 2.3% of the U.S. gas production^14^ and a significant fraction (over 30%) of the entire anthropogenic U.S. CH_4_ emissions^15^.

Given the considerable climate impact from CH_4_ emissions and the sizable proportion of emissions from the oil/natural gas supply chain, reducing CH_4_ emissions from this sector is being seriously discussed, particularly in the U.S., where the White House announced the goal to reduce national greenhouse gas emissions by 50 to 52% below its 2005 emissions levels by 2030 and specifically mentioned “methane abatement” as one of the strategies^16^.

To inform policymakers about CH_4_ emissions and the response of emissions to policy levers, a quantitative understanding of emissions and their change over time is paramount. However, despite considerable insights from past research into CH_4_ emissions from the oil/gas supply chain, an understanding of changes in emissions from year to year and their long-term trends over multiple years remains elusive, with widely divergent trends in U.S. emissions derived by different studies^17–21^.

The difficulty in understanding long-term emissions from the oil and gas sector stems in large part from observational limitations. Direct on-site surveys of oil/gas facilities can identify component-level emissions^22^, but requires special safety considerations and operator permission. Mobile methods have been developed that estimate emissions from entire well pads using roadside atmospheric plume measurements^23–25^. The aforementioned observational methods provide important insight at the scale of individual facilities, but the methods are labor intensive and difficult to sustain continuously over multiple years.

Moreover, the fact that emissions are dominated by quasi-stochastic emissions from a small fraction of locations due to abnormal conditions^12,14^ imposes severe sampling requirements for facility-level measurement techniques. Large emissions that only occur sporadically in a small number of facilities can be easily missed by labor-intensive techniques that sample individual facilities one by one.

Alternatively, the atmosphere can serve as an integrator of emissions from an entire distribution of facilities within an oil/gas production basin, manifested as enhancements in CH_4_ concentrations within the atmosphere. Such “top-down”, atmosphere-based estimation methods have included aircraft mass balance methods that estimate regional emissions from the change in CH_4_ content upwind versus downwind of the oil/gas production region^26–28^. Airborne remote sensing can quantify emissions from plumes emitted by numerous point sources over regional scales, demonstrating great promise in identifying super-emitters^29^. Nonetheless, while of great value, aircraft-based mass balance or remote sensing methods have provided mostly temporal snapshots of basin-wide emissions due to the associated resource and labor requirements.

Space-based top-down methods have also recently shown promise for estimating oil/gas emissions, particularly from the TROPOMI instrument on board the European Space Agency’s Sentinel 5 Precursor satellite^30–32^. Nonetheless, TROPOMI sampling of CH_4_ is limited by clouds, high terrain, and sensitivity to surface albedo^33,34^. Over high terrain, steep slopes, and the ocean, CH_4_ retrievals with appropriate quality assurances are currently unavailable^35,36^. Most importantly, TROPOMI CH_4_ observations are only available starting from the latter part of 2018, precluding a long-term analysis.

Long-term in-situ atmospheric monitoring possesses a long legacy in providing valuable scientific insights into the trends and spatial patterns of emissions in various target species, including greenhouse gases^37–39^. Yet most long-term in-situ CH_4_ observations in the U.S., with minor exceptions^40^, are sited in locations designed to characterize large-scale tracer gradients^41^, far away from the oil/gas production regions^20,42^, thereby reducing their sensitivity to oil/gas emissions.

**Figure 1:**
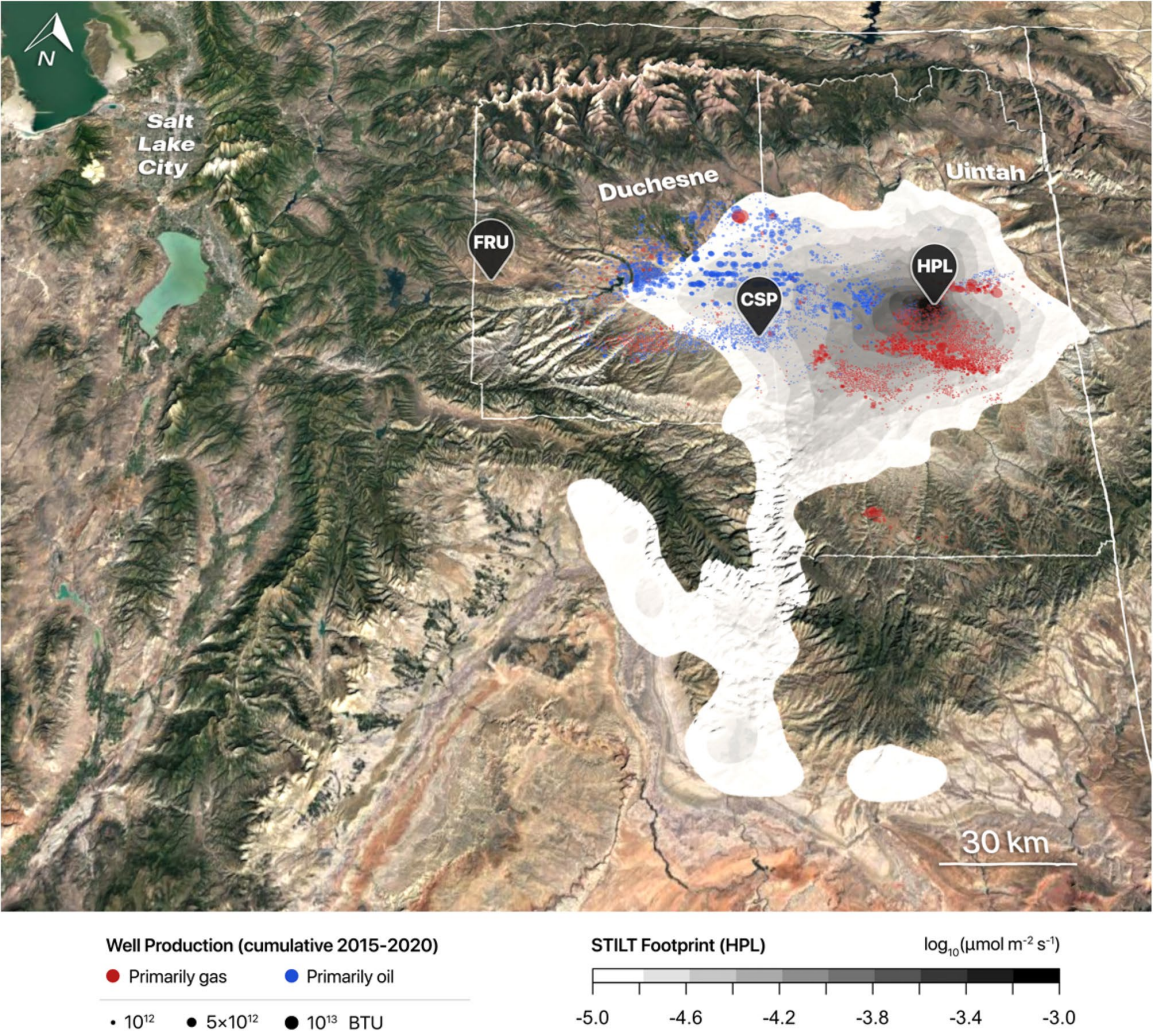
Map of the Uinta Basin in eastern Utah, CH_4_ observational sites, and oil/gas wells. Observational sites indicated by the black pins are: Horsepool (HPL), Castlepeak (CSP), and Fruitland (FRU). Locations of wells are indicated by circles, respectively. The well sizes reflect the cumulative production of energy (in [Btu]) from the sum of both natural gas and oil, using conversion factors described in the “Methods” section. If more energy is produced from natural gas, the well is colored in red, while wells producing more energy from oil is colored in blue. The east–west partitioning of gas versus oil wells also correspond geographically to the Uintah and Duchesne counties, which are separately dominated by gas and oil wells, respectively. The grayscale is the atmospheric footprint of the HPL site [ppm/(μmole m^−2^ s^−1^)] as simulated by HRRR-STILT, averaged over the subset of months (Apr to Sep) and afternoon hours (13:00 to 16:00 MST) from 2015 to 2020 used to calculate CH_4_ emissions. The underlying satellite image was generated from Google Earth, with data from: Landsat/Copernicus, TerraMetrics, Google.

**Figure 2:**
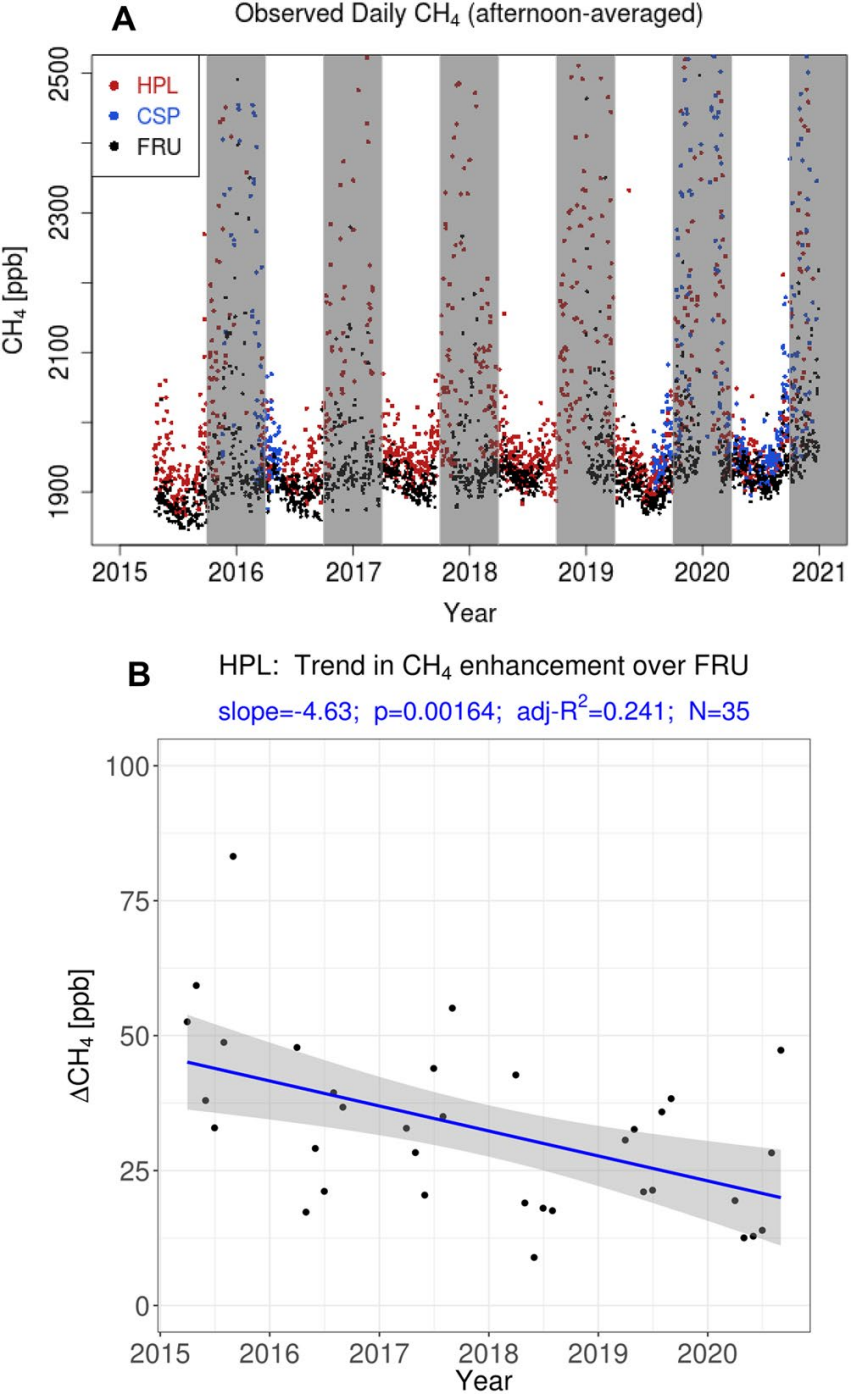
Time series of observed CH_4_ time series in the Uinta Basin between 2015 and 2020. (**A**) Observed daily CH_4_ averaged over afternoon hours (13:00 to 16:00 MST) at the HPL, CSP, and FRU sites. Gray regions cover the cold season spanning the months of Oct to Mar, when CH_4_ values can rise dramatically at especially HPL and CSP to > 3000 ppb (off-scale), especially during stable, cold air pool conditions that are difficult for atmospheric models to simulate with fidelity. To filter out the cold air pool conditions, a subset of months from Apr to Sep are chosen for analysis with the atmospheric model in order to calculate CH_4_ emissions. (**B**) Observed afternoon enhancements in CH_4_ at HPL over the baseline observed at FRU, averaged monthly from 2015 to 2020. The decreasing trend line derives from an ordinary least square regression; the resulting slope, *p*-value, adjusted-R^2^, and number of points (*N*) are shown in blue. Gray shading indicates 95% confidence interval of the fitted trend line.

Here we present a unique analysis of a multi-year record of in-situ CH_4_ observations from multiple sites within the Uinta Basin, an oil/gas production basin in eastern Utah (Fig. 1). Started in 2015, the Uinta Basin CH_4_ observations (Fig. 2) comprise the longest continuous in-situ CH_4_ record in an oil/gas production region, to our knowledge. Combining the multi-year in-situ observations with atmospheric modeling, we address two key questions: First, what information do long-term measurements within an oil/gas production basin provide? Second, how have CH_4_ emissions changed over multiple years?

The in-situ observations contain information from a broad swath of the oil/gas production region of the Uinta Basin (Fig. 1), reflecting emissions at spatial scales that are comparable to the mass balance aircraft technique, sustained over multiple years. Based on these observations, we show CH_4_ emissions from the Uinta Basin to track natural gas production, decreasing over multiple years as gas production declines from a peak in the first half of the 2010s. The decreases in CH_4_ emissions took place even before the COVID-19 pandemic. As a percentage of natural gas production, however, the relative emission rate remained steady between 6–8%, a level that is among the highest in the U.S.^14^. The emissions translate to 3–5% of the total energy produced from the Uinta Basin—from both oil and natural gas—being lost as CH_4_ to the atmosphere. An effort to reduce such CH_4_ leaks from the oil/gas infrastructure would not only yield climate benefits, but could also help the energy industry recoup at least some of its cost invested in leak detection and repair by recovering an economically valuable product^43,44^.

## Results

### CH_4_ emission trends

**Figure 3:**
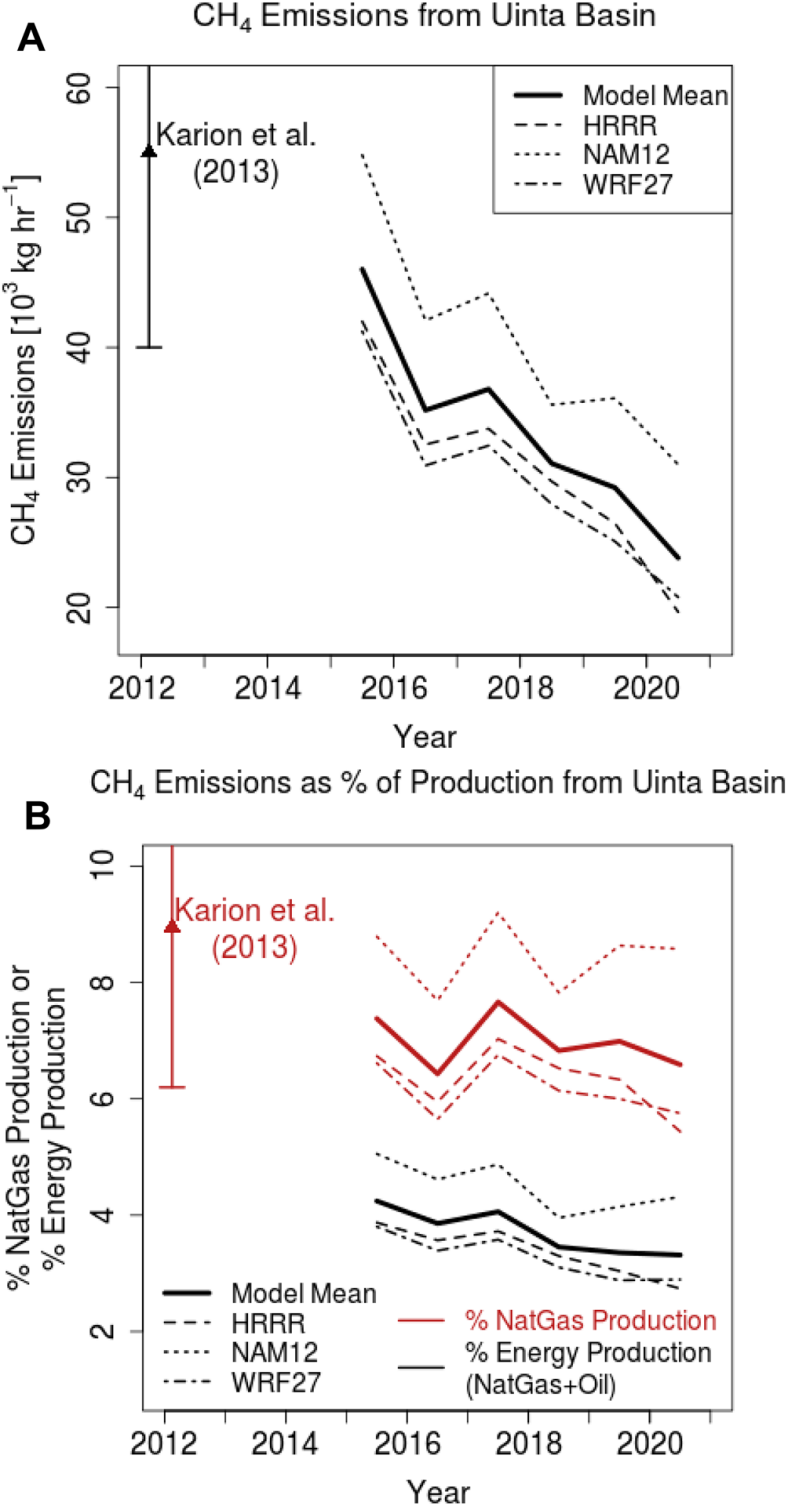
Annual CH_4_ emissions from the Uinta Basin. (**A**) Multi-year CH_4_ emissions calculated from CH_4_ enhancements observed at the HPL site (Fig. 2B) and atmospheric modeling (Eq. (1) in “Methods”). Multiple meteorological fields used to drive the STILT model are indicated by the three different dashed lines. The thick solid line is an average of results calculated from the three meteorological fields. Results based on Feb 2012 aircraft mass balance analyses from Karion et al.^26^ are also shown, with error bars (1-*σ*). (**B**) Multi-year CH_4_ emissions shown as percentage of the natural gas produced from the Uinta Basin (red) or as percentage of the total energy produced (black; including both natural gas and oil). Dashed lines and thick solid line, similar to (A), again refer to results from different meteorological fields and the multi-model average, respectively.

CH_4_ emissions derived from combining observed CH_4_ enhancements (∆*CH*_*4*_; Fig. 2) with atmospheric modeling (see “Methods”) indicate a decline from 2015 to 2020 that approximately halved emissions over this period (Fig. 3A). The emissions decline is robust, holding across three meteorological data products used to drive the atmospheric model. The emissions drop reflects the decrease in observed ∆*CH*_*4*_ (Fig. 2B) rather than trends in atmospheric transport and mixing (Fig. S2). During the early part of the observational record, CH_4_ emissions were higher, close to the range of 55 ± 15 × 10^3^ kg h^−1^ based on mass balance aircraft flights in 2012^26^ and later corroborated by an analysis of the 2015–2016 in-situ data^45,46^.

**Figure 4:**
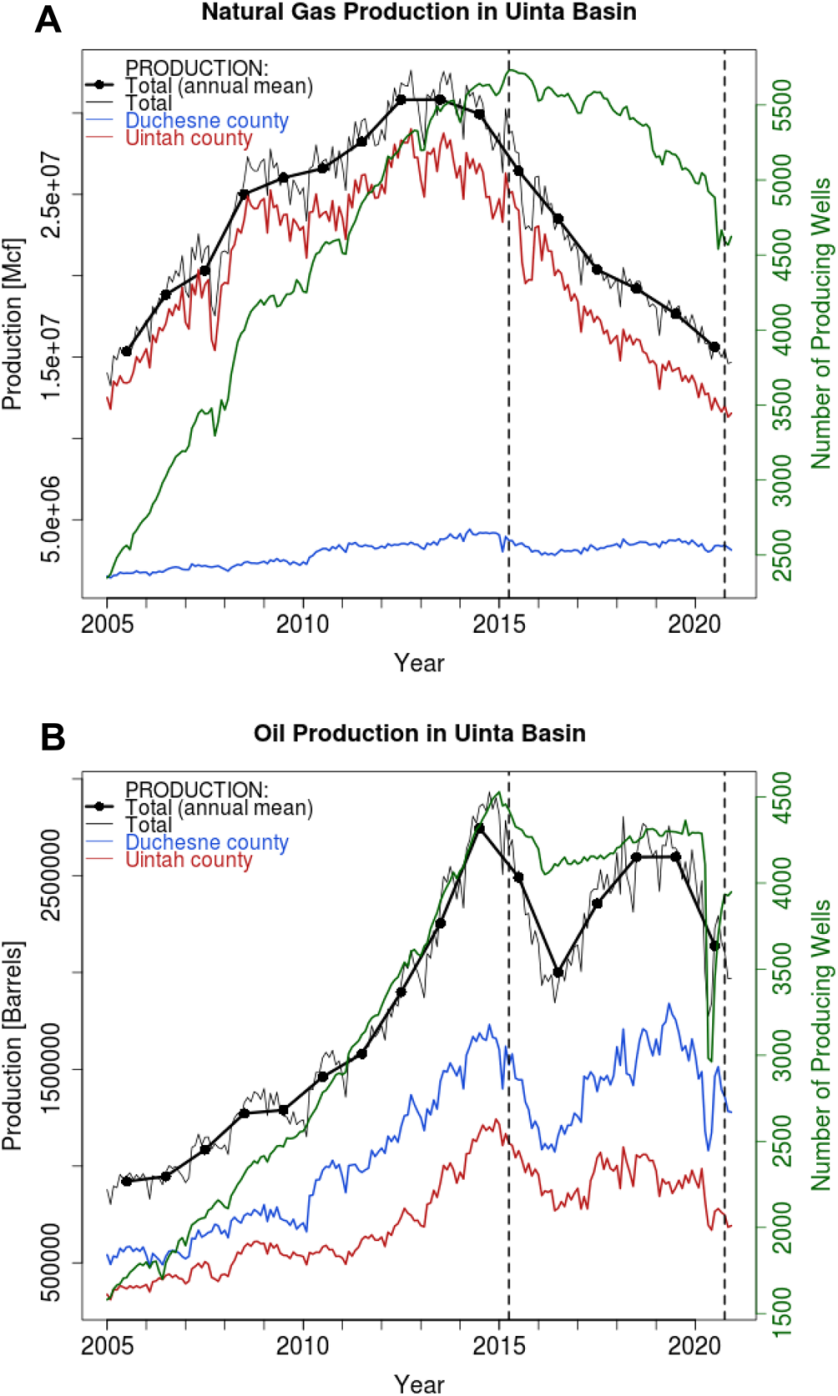
Natural gas or oil production in the Uinta Basin. (**A**) Natural gas production from the Uinta Basin between the years of 2005 and 2020, in units of one thousand cubic feet [Mcf]. The thick black line is the annual mean, while the thin black line is the monthly value. The monthly value is further broken down into production from the Uintah (red) and Duchesne (blue) counties, dominated by gas and oil wells, respectively (Fig. 1). The number of producing gas wells over the same period is in green. The six-year period overlapping with the in-situ CH_4_ analysis is between the dashed vertical lines. (**B**) Similar to (**A**), but for oil production in [Barrels] and the number of producing oil wells (green).

During the same six-year period in which CH_4_ emissions roughly halved, natural gas production in the Basin also decreased substantially to almost half, from 3.2 × 10^8^ Mcf in 2015 to 1.9 × 10^8^ Mcf in 2020 (Fig. 4A) as drilling activity declined after a collapse in fossil fuel prices after 2014^47^. The decline in natural gas production in 2020 did not appear to be any more pronounced than previous years, even during the mid-2020 COVID-19 shutdown period.

### Leakage rate

Declining CH_4_ emissions coinciding with decreasing natural gas production meant that expressed as a percentage of natural gas production (aka “leakage rate”), relative CH_4_ emissions remained steady over the six years, at ~ 6% to 8% of the produced natural gas, with the value depending on the driving meteorology adopted in the atmospheric model (Fig. 3B).

In contrast to natural gas production, oil production during the same period did not exhibit a long-term decline, first dropping in 2016, recovering by 2019 before declining again in 2020 (Fig. 4B). The COVID-19 shutdown period of May–June 2020 showed up as a particularly sharp decline in oil production that also coincided with a crash in oil price, which briefly dipped below zero^48^.

Expressing the CH_4_ emissions as a percentage of the total energy produced within the Uinta Basin from both oil and natural gas as the Energy-Normalized Methane Average (ENMA)^25^, we see that the CH_4_ emissions represent 3 to 5% of the total energy. This percentage of energy is being lost as CH_4_ emitted to the atmosphere.

### Relationship to well characteristics

The long-term in-situ observations enabled observed ∆*CH*_*4*_ to be related to well characteristics in the source regions (Figs. S3, S4), as informed by the “atmospheric footprint” (Fig. 1; “Methods”). The spatial scale of the source region contributing to in-situ observations is much larger than individual wells, so ∆*CH*_*4*_ is related to the aggregate influence of multiple wells. The multi-year CH_4_ emissions calculated in this study (Fig. 3) are based on the longer record from the HPL site, so the source region is weighted more heavily towards the eastern portion of the Basin, where gas wells dominate (Fig. 1). This region is also where a significant fraction of emissions in the Basin is expected, as previous isotope measurements have revealed the gas wells to be the predominant source (85%) of CH_4_ in the Uinta Basin^49^.

After convolving the well data with the simulated footprint to account for atmospheric transport, observed ∆*CH*_*4*_ is positively correlated with well characteristics, including the production amount of natural gas or oil and the density of gas- or oil-producing wells (Supplement). For the HPL site, the correlation is higher for natural gas-related variables (Fig. S3), with the Pearson correlation exceeding 0.5 and similar in value between gas production and density of producing gas wells, since the spatial distribution of natural gas production is itself strongly correlated with the presence of producing wells. Several studies of well-level measurements have found CH_4_ emissions to be positively correlated with gas production^22–24,50^.

The slope of ∆*CH*_*4*_ versus footprint-convolved natural gas production is an independent estimate of the percentage emissions from natural gas production (see “Methods”). The derived leakage rate was 6.3 ± 0.3% (± 1*σ*), over the six years of observations at HPL (Fig. S3), corroborating the leakage rate of 6.3 ± 0.6% for results from the same HRRR meteorology (Fig. 3B) determined alternatively using Eq. (1) in the “Methods” section.

At CSP, in proximity to oil wells in Duchesne county (Fig. 1), the correlation strength between ∆*CH*_*4*_ and natural gas production was comparable to the correlation against oil production and the producing oil wells (Fig. S4). The derived leakage rate from the slope of ∆*CH*_*4*_ versus footprint-convolved natural gas production at CSP is much higher, at 14.9 ± 1.2%. A previous study in the Uinta Basin also found much higher leakage rates from oil wells than from gas wells^25^. However, the oil wells produce only 22.5% of the natural gas in the Basin, on average, during the 2015–2020 period, so we expect the Basin-wide CH_4_ emissions calculated in this study (Fig. 3A) to reflect more contributions from the gas wells. Isotope measurements in 2013 have pointed to oil wells contributing only approximately 15% of the Basin-wide CH_4_ emissions^49^. Regardless, the contrast between oil versus gas wells underscores the heterogeneity in CH_4_ emissions within the Basin that are being mixed into the atmosphere and reflected in the enhancements in atmospheric CH_4_.

## Discussion

Based on one of the longest-running in-situ CH_4_ records in an oil/gas production basin, we found decreasing CH_4_ emissions from 2015 to 2020 that tracked the decline in natural gas production (Figs. 3A, 4A). From previous studies, CH_4_ emissions in oil/gas production regions have been traced to a variety of sources: storage tanks^13,51^, dehydrators^22^, liquid unloading^52^, gathering stations^53^, pneumatic controllers^54^, or other equipment such as connectors or pipelines^55^. Higher gas production has been suggested to subject the aforementioned equipment to more throughput and thus higher pressure, thereby leading to CH_4_ emissions that track production^56^.

The atmospheric footprints of the in-situ sites sample the integrated contributions from a variety of sources, and the atmospheric observations suggest that the portion of CH_4_ emitted to the atmosphere relative to the natural gas production held steady, between 6 to 8% (Fig. 3B). The fact that the leakage rate did not change appreciably over six years, despite a large decrease in natural gas production, was somewhat surprising. As gas production decreases, one could expect the leakage rate to rise, as previous research has found wells with lower production to emit a larger proportion of produced gas^50^.

While some regulatory measures were in place during the 2015–2020 time period, regulation likely did not play a significant role in CH_4_ emission reductions. The U.S. Environmental Protection Agency issued regulations governing organic compound emissions between October 2012 and October 2015 and then rules requiring natural gas leak inspection and repair in 2016^47^. However, these regulations applied solely to new oil and gas facilities, which were limited in number in the Uinta Basin as drilling slowed down between 2015 and 2020. In fact, the total number of wells in production decreased somewhat during the six years (Fig. 4).

While we are not sure why the leakage rate remained constant, we speculate that voluntary measures by some industry players may have had an influence. Surveys of a few companies operating in the Uinta Basin have revealed one instance of a company voluntarily installing equipment and carrying out leak detection/repair, resulting in fewer detected emission plumes^56^. How widespread these voluntary measures were adopted by other companies remains unknown.

CH_4_ emitted to the atmosphere amounted to as much as 3 to 5% of the total energy produced from the Basin (Fig. 3B). This non-negligible fraction suggests that efforts invested in detecting and fixing leaks could yield not just immediate climate benefits^7^, but also economic dividends. A recent analysis from the International Energy Agency demonstrates leak detection/repair could help recoup the cost, if only partially, for the energy industry in general^44^ due to the financial value of additionally recovered natural gas. Previous studies have already indicated that significant emission reductions are feasible from reducing leaks by detection efforts and equipment replacement^14,56^.

Natural gas is likely to continue serving a key role in the global energy mix over the next several decades even under decarbonization plans^57^. Thus, reductions in the leakage rate are necessary in order to decrease fugitive CH_4_ emissions without a simultaneous need to lower natural gas production. Given the White House goal of reducing the U.S. greenhouse gas emissions by 50% to 52% below its 2005 emissions levels by 2030^16^, addressing methane leaks is a measure that warrants immediate consideration.

Furthermore, leak detection and repair could potentially yield air quality benefits. Non-methane organic compounds, precursors of tropospheric ozone, are co-emitted with CH_4_ from oil/gas operations^51,56^, as seen in CH_4_ levels being strongly correlated with other organic compounds in the Uinta Basin^58,59^. The emissions of ozone-precursors in addition to CH_4_, along with the higher leakage rate observed at CSP, the site strongly influenced by oil production (Fig. S4), means that there is a need for leak detection and repair in oil wells as well as gas wells. Reductions in CH_4_ emissions would likely reduce levels of other organic compounds, thereby lowering ozone levels. This may already be taking place in the Uinta Basin, where wintertime ozone levels have decreased during the past decade^47^.

Since the results presented in this study characterize just one of the several oil/gas production basins in the U.S., a valid argument could be made that the Uinta Basin may not be representative of the U.S. as a whole. The Uinta leakage rate of 6 to 8% is one of the highest in the U.S. and is much higher than the national average of ~ 2%^14^, which is due, at least in part, to an abundance of low-producing wells in the Uinta Basin^50^. Considering the information yielded by in-situ observations shown in this study and the need to track trends in methane emissions to assess progress towards U.S. emission goals, this study demonstrates an urgent need to establish more in-situ, long-term CH_4_ observations in other oil/gas production basins to complement satellite-based monitoring and emissions inventory efforts^41^.

## Methods

### Uinta basin

The Uinta Basin is a well-defined mountain basin in eastern Utah (Fig. 1), bordered by the Uinta Mountains to the north, the Tavaputs Plateau to the south, and the Wasatch Range to the west. Oil/gas production dominates the economic activity in the Uinta Basin, a rural area where population is about 50,000 and agriculture is limited^60^. The Uinta Basin produces considerable amounts of oil per unit of natural gas^25^. The approximately ~ 10,000 producing wells in the Uinta Basin (Fig. 4) exhibit an east–west contrast, with the Green River serving as a rough demarcation line (Fig. 1). Gas wells are mostly found east of the river, in the east/southeastern corner of the Basin in Uintah County, while oil wells dominate in the west/northwest, mostly in Duchesne County.

Production of both natural gas and oil from the Uinta Basin increased rapidly in the 2000s, following the widespread adoption of horizontal drilling and hydraulic fracturing nationwide (Fig. 4). Natural gas production peaked in 2013/2014 and continued declining thereafter, as drilling activity also decreased^47^. Oil production also peaked in 2014, and dipped sharply in 2016 but recovered in 2017–2019 before decreasing again in 2020, reflecting the dramatic collapse of oil prices in mid-2020^48^.

Oil/gas production activities emit other organic compounds in addition to methane. Many of these compounds react chemically in the shallow cold air pools that form in the confined airshed created by the terrain of the Uinta Basin^61^, allowing ozone to be rapidly produced during some winters, particularly in the presence of snow-cover. During such conditions, ozone levels can exceed the National Ambient Air Quality Standard^59,60^, putting the Uinta Basin in marginal nonattainment status.

### Observational sites

The CH_4_ observational sites discussed in this study are shown in Fig. 1. The Fruitland site (FRU; 40.209^o^N, 110.840^o^W) is on the western (generally upwind) edge of the Uinta Basin, at 2024 m above sea level and ~ 400 m higher in elevation above the other two sites. As such, FRU serves as a background site^45^ characterizing baseline levels of CH_4_ in the absence of influence from oil/gas production (Fig. 3A). The Castlepeak site (CSP; 40.051^o^N, 110.019^o^W) is in the midst of the oil wells in Duchesne county and was installed temporarily between November 2015 and May 2016 before long-term installation started in August 2019. The Horsepool site (HPL; 40.143^o^N, 109.468^o^W) is the longest running site found in Uintah county in the eastern part of the Basin, near both gas and oil wells. The HPL record started in February 2015 and continues to the present day. A fourth site, in the town of Roosevelt (ROO), was terminated in July 2019 and excluded in the present analysis due to the combination of a weaker emission signal and the influence of a highly localized well near the site^45^.

CH_4_, along with CO_2_ and H_2_O, are measured at each site via the Off-Axis Integrated Cavity Output Spectroscopy (OA-ICOS) technique using a Los Gatos Research Ultraportable Greenhouse Gas Analyzer (907-0011, Los Gatos Research Inc., San Jose, CA). Measurements are recorded as 10-s integrations of 1-Hz observations, which are then averaged to hourly intervals. The instruments are calibrated every 3 h using three dry air gas cylinders traceable to World Meteorological Organization (WMO) standards maintained at the National Oceanic and Atmospheric Administration (NOAA). Typical uncertainties in the CH_4_ measurements are ~ 3 ppb^62^. For more details regarding the instrumentation and data quality, see Bares et al.^62^.

The observed afternoon-averaged CH_4_ time series exhibit a distinct seasonal pattern, with positive excursions reaching hundreds to even thousands of ppb between the months of October and March (shaded in Fig. 2A), especially at HPL and CSP. The extreme enhancements reflect suppressed mixing and buildup of CH_4_ during cold air pools^31,46^. Previous researchers have alluded to the difficulties atmospheric models face in reproducing the cold air pools and the associated atmospheric transport^61,63^. We took advantage of the long measurement record and only focused on the months of April to September in each year to avoid emission estimates being subject to large errors in atmospheric modeling (see below). Similarly, elevated CH_4_ levels are also observed during the nighttime (Fig. S1) when emissions accumulate within the stable nocturnal boundary layer, which likewise can be difficult to simulate with atmospheric models^59^. Consequently, we focus on analyses of afternoon observations, when well-developed convective mixing takes place^45^. The four hours spanning 13:00–16:00 MST (20:00–23:00 UTC) were selected to represent the afternoon; these are the same hours as selected in a previous analysis of the Uinta Basin CH_4_ data^46^.

### Atmospheric modeling

To estimate CH_4_ emissions from observed CH_4_ mixing ratios, atmospheric simulations were carried out based on the Lagrangian Particle Dispersion Modeling technique^64^. An ensemble of air parcels was released at the HPL and CSP sites within the Stochastic Time-Inverted Lagrangian Transport (STILT) model^65^ and advected backward in time. The air parcels incorporate the effects of both turbulent dispersion and grid-resolved winds, both derived from driving meteorological fields. As the air parcels transport backward in time and disperse, they trace upstream the trajectories of air arriving at the observational sites. The air parcel trajectories elucidate the source region of the observational site and the sensitivity of mixing ratio observations at the site and upwind emissions—also referred to as the “atmospheric footprint”^65^, in units of [ppm/(μmole m^−2^ s^−1^)]. Here the footprint was resolved at 0.01° × 0.01° spacing.

The trajectories of 200 air parcels were simulated every afternoon hour from 13:00 to 16:00 MST for the entire observational record from 2015 to 2020 and traced backwards in time for 24 h. An earlier analysis found more than 99.5% of air parcels released from HPL to exit the Uinta Basin within 24 h^45^. A sensitivity analysis using an order-of-magnitude larger ensemble size of 2000 parcels resulted in annual emissions different by only 4% (Supplement), a small random deviation compared to the much larger difference between meteorological drivers (details below). Kernel density estimation was used to calculate atmospheric footprints based on locations of air parcels from a limited ensemble size, thus reducing the sensitivity to ensemble size^66^.

The latest version of the STILT model adopted here represents a merger of STILT features with those from NOAA’S HYSPLIT model to take advantage of advances in the code base^67^. The base STILT simulation was driven by meteorological fields from NOAA’s High-Resolution Rapid Refresh (HRRR) system^68^, at 3-km grid spacing and hourly frequency, downloaded from the NOAA-Air Resources Laboratory. The average footprint of the HPL site averaged is shown in Fig. 1. We see that the footprint sensitivity for HPL covers gas wells to the south as well as oil wells to the west, with most of the footprint found within the Uinta Basin, suggesting that CH_4_ measured at the HPL site contains information about emissions from a wide swath of the Basin. Following this logic, we estimate the Basin-averaged CH_4_ emissions Φ by dividing the CH_4_ enhancement measured at HPL by the total footprint integrating over all gridcells *i* within the Uinta Basin (defined as between 39.9^o^N to 40.5^o^N and 110.6^o^W to 109^o^W):

1 $$\begin{aligned} & \Phi = \frac{{\Delta CH_{4} }}{{\sum\nolimits_{i} {f_{i} } }} \\ & \Delta CH_{4} = CH_{{4HPL}} - CH_{{4FRU}} \\ \end{aligned}$$

where Φ at daily timescales was determined by dividing the ∆*CH*_*4*_ averaged over the afternoon (13:00–16:00 MST) by the total footprint averaged over the same hours. Gaps in the baseline value measured at the FRU site (*CH*_4*FRU*_) were filled using monthly averages. Sensitivity tests revealed that removing the gap filling had a minimal effect on the annual emissions and trend (Fig. S7), likely because the sub-monthly variability at FRU was low to begin with, and averaging over multiple months further reduced the sensitivity to gap filling.

Equation (1) calculates the average CH_4_ emissions Φ from a unit area within the Basin that would account for the observed CH_4_ enhancement, given the transport pathway and residence time of air as simulated by STILT. ∆*CH*_*4*_ is in units of [ppm], while footprint *f*_*i*_ is in [ppm/(μmole m^−2^ s^−1^)], so Φ, the quotient between the two quantities, is in flux units of [μmole m^−2^ s^−1^]. Φ is then multiplied by the area of the Uinta Basin to arrive at Basin-wide emissions. A similar approach has also been adopted by other researchers to estimate CH_4_ emissions in Alaska^69,70^. A sensitivity analysis limiting the domain over which the footprint is summed to the eastern portion of the Basin—the Uintah County—produced similar Basin-wide emissions when emissions from the western portion of the Basin were accounted with other means (Fig. S8).

We filtered out simulated times when $\sum\limits_{i} {f_{i} }$ was smaller than its 10th percentile value, indicating low sensitivity to surface emissions within the Basin. A threshold of 10 days was set for a monthly averaged Φ to be retained in determining the annual average, determined from the average of the months from April to September (see above). Only two months (May 2016 and Sep 2018) were removed from analyses due to this criterion.

*f*_*i*_, as a modeled quantity, is sensitive to errors in the HRRR-STILT simulation. We addressed potential errors in HRRR by comparing wind vectors extracted from HRRR against hourly-averaged wind observations at the HPL and CSP sites, downloaded from the Mesowest database^71^. Whenever measured windspeeds exceeded 1 m s^−1^, indicating times when wind direction was well-defined, these times were removed from analysis if the horizontal wind direction in HRRR deviated from the observed by ± 45°. This filtering removed 35% of the hours. A sensitivity analysis showed that wind-based filtering did not appreciably affect results (Figs. S5, S6).

We further tested the sensitivity of the calculated Φ to meteorological fields, driving STILT with the NAM (12-km) and WRF (27-km) fields, also downloaded from the NOAA-Air Resources Laboratory. All three models exhibited similar qualitative patterns, showing declining annual emissions along with a relatively constant leakage rate (Fig. 3). However, the magnitude of the emissions differed between meteorological models, with NAM resulting in significantly higher emissions than HRRR or WRF. We suspect the differences can be traced to differing rates of vertical exchange between the boundary layer and the free troposphere affecting $\sum\limits_{i} {f_{i} }$, with $\sum\limits_{i} {f_{i} }$ being noticeably lower in NAM than the other two models (Fig. S2). Due to the differences between the three models, we report the range of the modeled results as a rough measure of uncertainties in derived CH_4_ emissions.

### Trends in meteorology

To test the hypothesis whether trends in meteorology could account for the decreased emission trends, we examined trends in Basin-summed footprint strengths ($\sum\limits_{i} {f_{i} }$) using the HRRR, NAM, and WRF fields. No significant trend was detected for the years between 2015 and 2020 for all three models (Fig. S2). The mid-latitude storm track typically moves north of Utah during much of the study period, decreasing the opportunities for variation in atmospheric conditions, while rainfall and rainfall variability are also typically small in the semi-arid Uinta Basin during the April – September months. We further examined trends in observed meteorology that could account for the emission trend using surface observations in the Uinta Basin (Fig. S9 and Tables S1-S4). Due to a lack of radiosonde data in the Basin domain, we constructed a “pseudo-lapse rate” using three surface weather stations at different elevations along a transect in the center of the Basin to probe changes in atmospheric stability (Fig. S9 and Table S1). The surface weather stations revealed no systematic trends in temperature, windspeeds, or pseudo-lapse rate between 2015 and 2020 (Tables S2-S4). See Supplement for more details. The lack of a trend in meteorology and resulting atmospheric transport points to the declining CH_4_ emissions simply arising from the decreasing ∆*CH*_*4*_ observed at HPL (Fig. 2B).

### Well data

Monthly well-level oil and gas production data were downloaded from the Utah Division of Oil, Gas and Mining (https://oilgas.ogm.utah.gov/). The well data were convolved with the STILT-simulated footprint to account for atmospheric transport linking the sources to the observed ∆*CH*_*4*_. Further normalizing by footprint gridcell area and the number of days in a month, the well data were used to create spatially explicit maps of footprint-convolved well density for the producing wells, in units of [well # × day^−1^ ppm/(μmole s^−1^)], oil production in [Barrels × day^−1^ ppm/(μmole s^−1^)], and gas production in [Mcf × day^-1^ ppm/(μmole s^−1^)]. These quantities were then summed for each day and related to the daily afternoon-averaged ∆*CH*_*4*_ observed at HPL to examine the potential relationship between ∆*CH*_*4*_ and well density or oil/gas production (Figs. S3, S4). The slope from linearly regressing ∆*CH*_*4*_ versus the footprint-convolved natural gas production is of particular interest, as the slope provides an alternative estimate of the leakage rate after accounting for the methane content of natural gas (below).

### Methane content of natural gas

Following Karion et al.^26^, the volume fraction of CH_4_ in natural gas was chosen to be 89%. A recent study^72^ sampled natural gas from wells in the Uinta Basin to determine the various hydrocarbons found in the samples. The study revealed that the volume fraction of CH_4_ in raw natural gas ranged from 80 to 91%. We decided to use the 89% value to maintain consistency with Karion et al.^26^, and 89% is towards the higher end of the range in CH_4_ content to provide a conservative (lower-end) estimate of the leakage rate. It is worth noting that if the lower end of the methane content (80%) was adopted, the leakage rate would be even higher than that shown in Fig. 3B.

### Energy-normalized methane average (ENMA)

The Energy-Normalized Methane Average (ENMA) emissions refer to the emissions of CH_4_ expressed as a percentage of energy produced from the entire Basin, in the form of both natural gas and oil. Thus, ENMA indicates the percentage of energy lost to the atmosphere from CH_4_ emissions. Following Robertson et al.^25^, we assumed 1 × 10^6^ Btu per Mcf of methane or natural gas and 5.8 × 10^6^ Btu per barrel of oil.

## Supplementary Information

Supplementary Information.

Supplementary Data 1.

Supplementary Data 2.

## Data Availability

“All data needed to evaluate the conclusions in the paper are present in the paper and/or the Supplementary Materials”.
