# Supplementary material for: Declining methane emissions and steady, high leakage rates observed over multiple years in a western US oil/gas production basin
Source: Sci Rep. 2021 Nov 16;11:22291. doi: 10.1038/s41598-021-01721-5 (PMC8595340; doi:10.1038/s41598-021-01721-5)
Supplement: Supplementary file 1 — Supplementary Information. [file 41598_2021_1721_MOESM1_ESM.docx]

Supplementary Materials for

**Declining Methane Emissions and Steady, High Leakage Rates Observed over Multiple Years in a western U.S. Oil/Gas Production Basin**

John C. Lin*, Ryan Bares, Ben Fasoli, Maria Garcia, Erik Crosman, Seth Lyman

*Corresponding author. Email: [John.Lin@utah.edu](mailto:John.Lin@utah.edu)

**This PDF file includes:**

Supplementary Text

Figs. S1 to S9

Tables S1 to S4

**Other Supplementary Materials for this manuscript include the following:**

Data files: UINTA_CH4.zip, Fch4.zip

Supplementary Text

Observed Diurnal Cycle

| 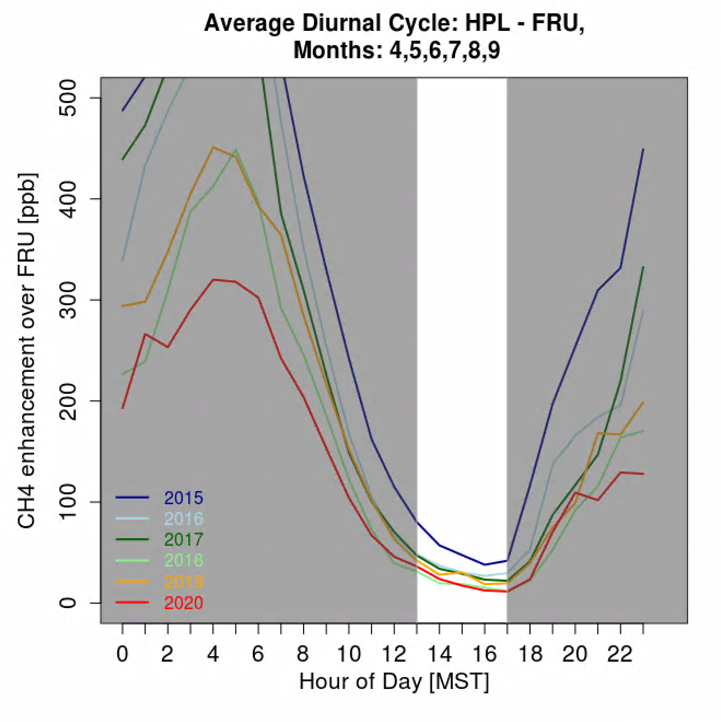 | 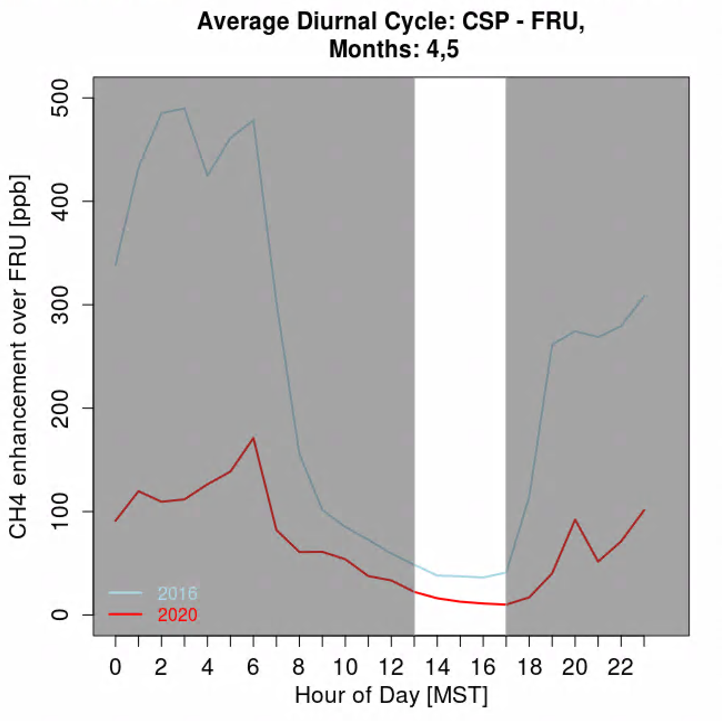 |
| --- | --- |

Fig. S1.

Annually-averaged diurnal cycle of observed CH_4_ enhancement (above the FRU baseline) at (Left:) HPL from 2015 to 2020 during the months from Apr to Sep and (Right:) at CSP in 2016 and 2020 during Apr and May. The hours of 13:00 to 16:00 MST (20:00 to 23:00 UTC) are selected to represent afternoon well-mixed conditions. The grayed hours are hours outside of 13:00 to 16:00 MST not incorporated in the analysis. The decline in CH_4_ enhancement over year years, shown in Fig. 2b, can also be seen here.

| 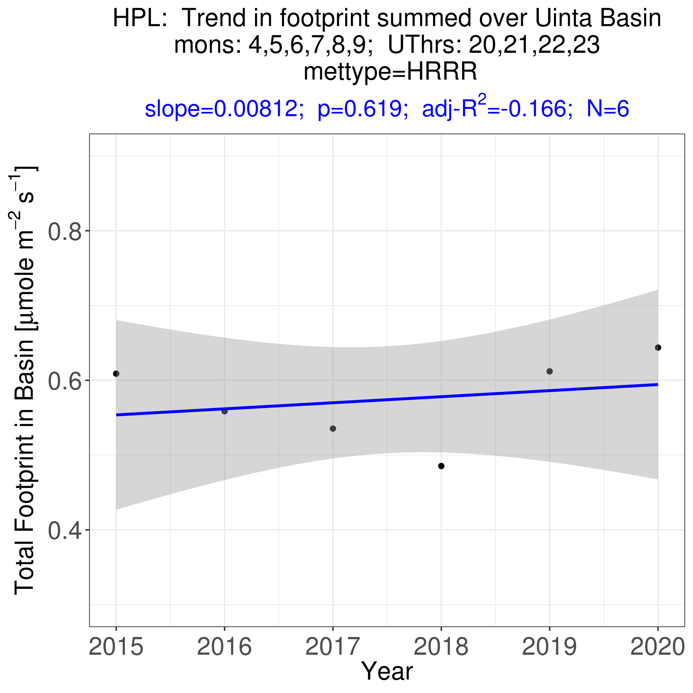 | 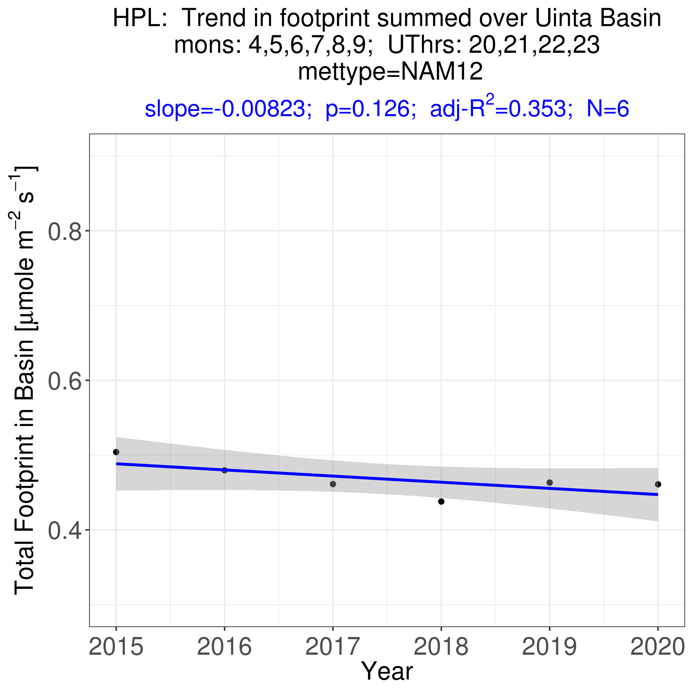 |
| --- | --- |
|  | 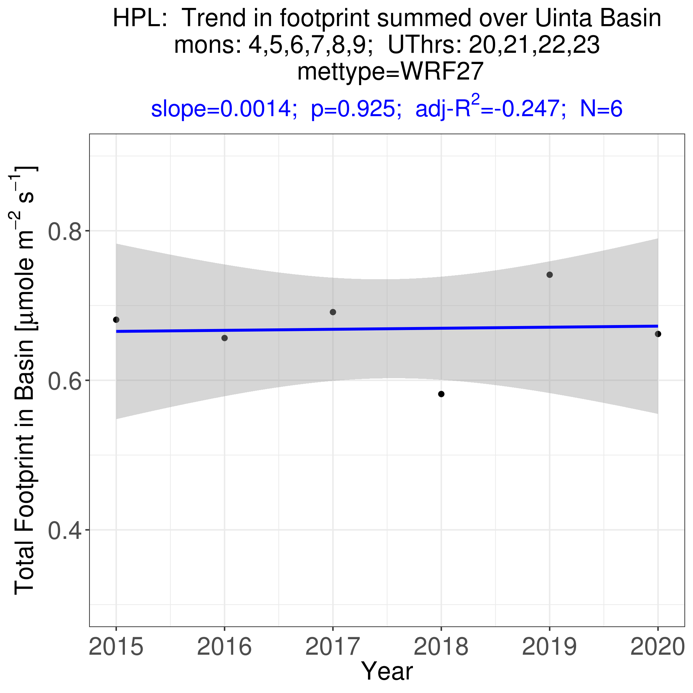 |

Simulated Footprint Trends

Fig. S2.

Annually-averaged footprint strength for the HPL site, summed over the Uinta Basin ($\sum_{i} f_{i}$), as simulated by STILT, driven with the HRRR (top-left), NAM-12 km (top-right), and WRF-27 km (bottom-right) meteorological fields. The trend line and the slope from fitting an ordinary least-square regression, *p*-value, adjusted-R^2^, and sample size (*N*) are shown in blue. The gray shading indicates the 95% confidence interval of the fitted trend line.

To examine whether the decreasing CH_4_ emission trend found using all three meteorological models (Fig. 3) can be attributed to trends in the Basin-summed footprint strengths ($\sum_{i} f_{i}$ in Eq. 1), we show in Fig. S2 the values of the average value of $\sum_{i} f_{i}$in each year, from 2015 to 2020, for the HRRR, NAM, and WRF models used to drive STILT. The trend in the Basin-summed footprint was statistically insignificant across all three models, with differing signs in the trends. This suggests that the declining CH_4_ emission trend was driven by decreasing trend in observed CH_4_ enhancement (Fig. 2b) rather than an increasing trend in $\sum_{i} f_{i}$.

|  |
| --- |

Relationship between Δ*CH_4_* and Well Characteristics

Fig. S3.

Linear relationships between the methane enhancement (Δ*CH_4_*) observed at the Horsepool (HPL) site and footprint-convolved well data. The atmospheric footprints were generated by driving STLIT with HRRR. Δ*CH_4_* is the observed enhancement over the FRU baseline site averaged for each day, during the afternoon hours (13:00-16:00 MST). The x-axes differing between the four panels include the gas production, in units of [Mcf × day^-1^ ppm/(μmole s^-1^)], oil production in [Barrels × day^-1^ ppm/(μmole s^-1^)], and density of producing wells, in units of [well # × day^-1^ ppm/(μmole s^-1^)], and. The slopes are derived from a standard major regression to account for errors in both the predictor and predictand (Δ*CH_4_*), after removing outliers (>99-th percentile) in both Δ*CH_4_* and the predictor variable. Errors in the slopes (1-σ) are calculated from repeating the regression for 1000 times, each time sampling the data with replacement (i.e., bootstrapping). The slope from linearly regressing Δ*CH_4_* versus the footprint-convolved natural gas production is of particular interest, since it provides another way to calculate the leakage rate [%].

|  |
| --- |

Fig. S4.

Same as Fig. S3, but for the Castlepeak (CSP) site.

Sensitivity Analyses

Here a series of sensitivity analyses are presented to examine the sensitivity of calculated CH_4_ emissions to various choices.

The base case is shown below in Fig. S5, which filters out times when the simulated wind direction in HRRR deviates from observed wind direction by ±45^o^ during times when windspeeds exceed 1 m s^-1^. Gaps in the FRU baseline are filled with the average observed afternoon values during the month. The subset of months, from April to September, are used to represent the annual average in order to avoid relying upon highly uncertain atmospheric simulations during the cold air pool events, when methane enhancements can reach hundreds or even several thousand ppb (Fig. 2a). Similarly, only the afternoon hours of 13:00-16:00 MST are used (Fig. S1), when the boundary layer tends to be well-mixed and atmospheric models are subject to smaller uncertainties.

| 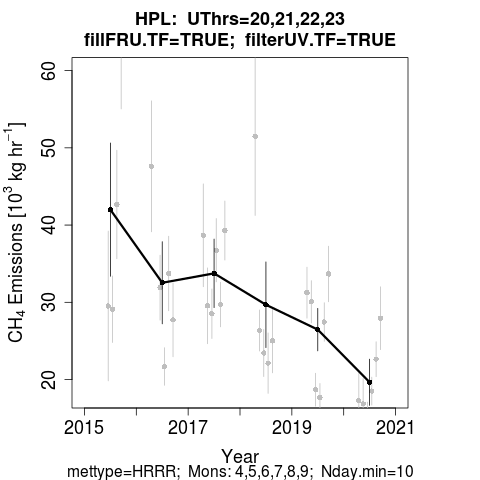 |
| --- |

Fig. S5.

Monthly-averaged (gray) and annual-averaged (black) estimates of CH_4_ emissions from the Uinta Basin from the methane enhancement observed at the HPL site and STILT-simulated atmospheric transport, driven with HRRR meteorology. Note that HRRR data are only available starting in June 15^th^, 2015. The error bars are the standard errors of the monthly or annual average, calculated by the day-to-day or month-to-month standard deviation divided by *N*^1/2^, where *N* is the number of days or months, respectively

Turning Off Transport Error Filtering

| 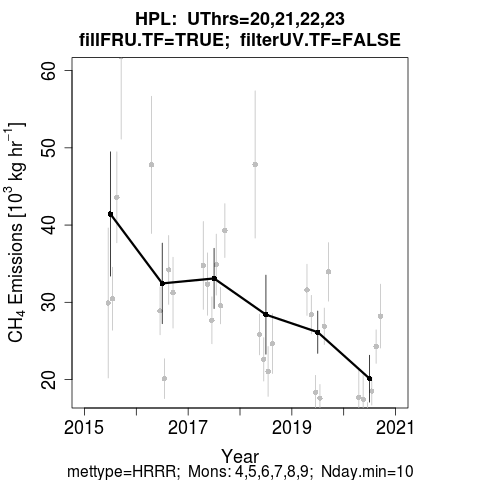 |
| --- |

Fig. S6.

Similar to Fig. S5, but without filtering out times when the simulated wind direction in HRRR deviates from observed values.

Comparing Figs. S6 and S5, we can see that removing the transport error-based filtering hardly altered the annual emission values.

FRU Background Not Gap-Filled

| 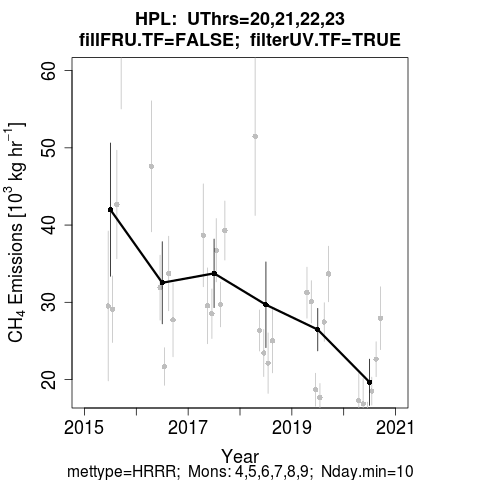 |
| --- |

Fig. S7.

Similar to Fig. S5, but without filling in gaps in the FRU time series with monthly averages.

The lack of gap-filling in the FRU baseline time series mostly resulted in annual emission patterns very similar to the baseline case (Fig. S5).

Changing Domain over which Footprint is Summed

A sensitivity analysis varying the gridcells over which the footprint is summed in the denominator of Eq. 1 was carried out. Instead of the entire Basin, the western edge of the domain was set at 110^o^W (instead of the original 110.6^o^W), near the boundary between the Duchesne and Uintah counties and just to the east of the CSP site (Fig. 1). This choice was made to limit the calculation domain to the Uintah County in order to focus on the main gas-producing region in the Basin. The latitude range remain unaltered, between 39.9^o^N to 40.5^o^N.

The comparison between the base case (also shown in Fig. S5) is shown as annual CH_4_ emissions below (Figs. S8). Since the altered domain covers only the Uintah County, the total emissions are lowered from the base case covering the entire Basin (both Duchesne and Uintah counties), due in large part to a smaller areal extent. To calculate the emissions from Duchesne county missing from the altered domain, the empirically-observed leak rate of 14.86% observed at the CSP site (Fig. S4) is applied to the gas production in Duchesne county, primarily from oil wells. After this correction is applied, the Basin-wide numbers between the Base case and the altered domain case are quite comparable, with subtle differences between the years. Thus, the domain change does not qualitatively affect the general decreasing trend in methane emissions, and the shift is within the range of the differences resulting between meteorological fields used to drive the STILT model (Fig. 3).

| 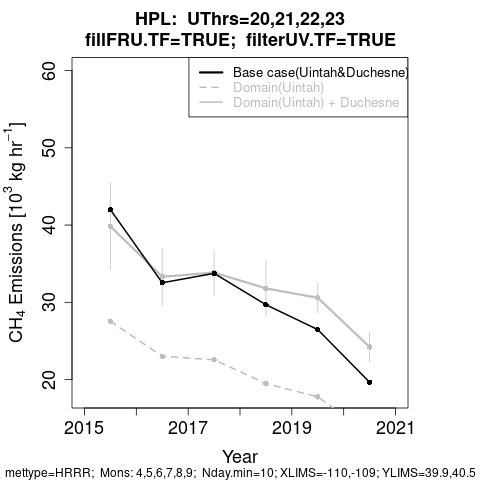 |
| --- |

Fig. S8.

Annual-averaged estimates of CH_4_ emissions from the Uinta Basin from the methane enhancement observed at the HPL site and STILT-simulated atmospheric transport, driven with HRRR meteorology. The base case is in black (also shown in Fig. S5), with model domain covering the entire Basin. The altered domain case covering only the Uintah county on the eastern part of the Basin is shown as dashed gray. The altered domain case with emissions from both Uintah county and Duchesne county, with the latter added using the empirically-observed leak rate at the CSP site (Fig. S4), is shown as gray.

Increasing Air Parcel Ensemble Size

The stochastic nature of air parcel motions within STILT means that simulation results differ slightly from run to run, and the statistical fluctuation is larger if the size of the parcel ensemble is smaller. To test the sensitivity of the calculated methane emission to ensemble size, we recalculated one year’s worth of methane emissions (from 2016) with an order-of-magnitude larger ensemble size of 2000, using HRRR as the meteorological field to drive STILT. The resulting emission for 2016 was 34.0 × 10^3^ kg hr^-1^, while the base case with 200 parcels (shown in Fig. S5) was 32.5 × 10^3^ kg hr^-1^, a 4% difference. Such a deviation was small compared to the much difference between the driving meteorology for STILT: NAM and WRF resulted in 42.1 × 10^3^ and 30.9 × 10^3^ kg hr^-1^, respectively.

Observed Meteorology Trends

In any air pollution trend analysis, the impacts of variations in the meteorological forcing – dry and wet pollution scavenging, transport and dilution of pollutants and their precursors through precipitation, and vertical and horizontal mixing processes, need to be accounted for. In this study, the interannual variations in meteorological forcing were small, likely mainly due to the storm track moving north of the Uinta Basin for most of the summer and early fall periods which dominated the analysis temporal span, as well only light precipitation observed across the semi-arid region. Our analysis of meteorological variability presented here will utilize inter-annual trend analysis of mean meteorological variables and derived pseudo-lapse rates as a proxy for variations in vertical mixing and stability for several weather stations in the Uinta Basin for the months of April, May, June, July, August and September from 2015 to 2020.

Meteorological Data and Proxies for Transport

Meteorological data was downloaded for the study period from three near-surface (10-m above ground level (AGL)) Remote Automatic Weather Stations (RAWS) through Mesowest ^1^. The location of the surface weather stations that were used are shown in Fig. S9. The station name, network organization, location, and elevation are listed in Table S1. The Fort Duchesne RAWS site is centrally located in the Uinta Basin at an elevation of 1502 m ASL, while Five Mile is located mid-slope (2280 m ASL), and Bruin Point near mountaintop on the Tavaputs Plateau, at an elevation of almost 3000 m ASL.

Meteorological reanalysis data from the National Center for Environmental Prediction (NCEP) and National Center for Atmospheric Research (NCAR) reanalysis product (NCEP/NCAR) of 700 hPa winds, 500 mb heights, and 2-m temperature were also obtained from the National Oceanic and Atmospheric Administration (NOAA) Physical Sciences Laboratory. Because these products corroborated the findings from the RAWS meteorological observations, they are not presented here.

Our analysis of meteorological variability included: 1) Variations in the 2-m temperature and 10-m wind speed between different years, as well as a 2) Pseudo-Lapse rate analysis. The Pseudo-Lapse rate analysis is used to estimate vertical mixing and hence pollution dispersion and transport rates in the boundary-layer in the absence of available vertical profiles of temperature. In the Uinta Basin, Pseudo-Lapse rate estimations have been applied previously (e.g., ^2^ ^3^). In addition, the use of a series of temperature sensors on terrain slopes to provide pseudo-profiles of the atmospheric temperatures has been also demonstrated in Utah’s Salt Lake Valley ^4^.

Table S1.

Remote Automatic Weather Stations (RAWS) weather stations analyzed in mid-basin elevation transect.

| Station Name | Network | Elevation (m) | Latitude °N/Longitude °W |
| --- | --- | --- | --- |
| Bruin Point | RAWS | 2973 | 39.609333/110.289972 |
| Five Mile | RAWS | 2280 | 39.890833/110.265833 |
| Fort Duchesne | RAWS | 1502 | 40.284772/109.861828 |

| 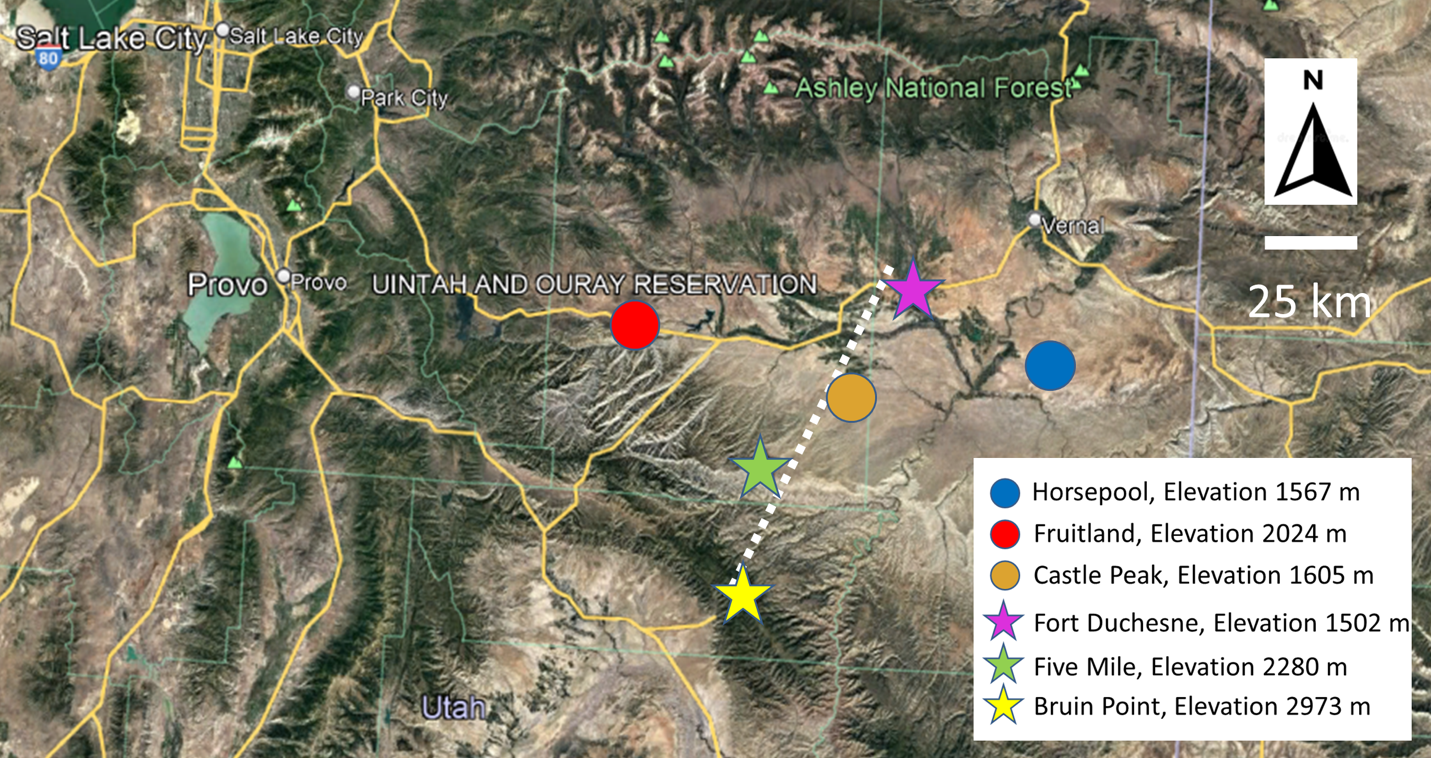 |
| --- |

Fig. S9.

Location of the three RAWS Sites listed in Table S1 (stars), along with the CH_4_ observational sites (circles). The white dashed line indicates the transect from low to high elevation. The underlying satellite image was generated from Google Earth, with data from: 2021 Google Image Landsat/Copernicus.

Meteorological Variability 2015-2020

The inter-annual variations in the April – September mean 2-m air temperatures observed at the three RAWS weather stations varied by 0.5 – 2.0 ℃ at the 3 locations, with 2015 being the coolest period, and 2018 the warmest (Table S2). The inter-annual variations in the average April – September 10-m wind speeds at the low and mid-slope locations (the high elevation location was removed due to suspected quality issues with wind speed) varied by less than 0.5 m s^-1^ at Fort Duchesne and ~1 m s^-1^ at Five Mile (mid-slope) (Table S3). The mean 700 mb zonal wind (mountaintop) anomalies compared to the 1981-2010 climatology were less than 1 m s^-1^ (not shown), demonstrating that variations in mean large-scale wind speed and transport were not an important driving factor in the inter-annual variations in CH_4_ observed.

The mean April - September Pseudo-Lapse rates (derived by calculating the differences in 2-m temperatures between locations at differing elevation) between the low, mid, and high elevation RAWS weather stations were fairly consistent between 2015 and 2020 (Table S2). The Pseudo-Lapse rates were very consistent between the mid and high elevation locations, with more variations in the lapse rate noted between the low and mid-elevation sites. The most stable lapse rate period noted between the low and mid-elevation location was observed in 2020 and the least in 2017, but these differences are relatively small (Table S2). Thus, the takeaway message is that changes in vertical stability from year to year between 2015 and 2020 are not expected to have a notable impact on the interannual variability of CH_4_ during the April -September time periods in the Uinta Basin.

Further restricting the mean April - September Pseudo-Lapse rates between the low, mid, and high elevation RAWS weather stations to only the afternoon hours of each day (13:00-16:00 MST ), which are the periods of interest utilized in this study, resulted in even less inter-annual variations in the mean yearly April - September Pseudo-Lapse rates between both low and mid-elevation and mid and high-elevation locations (Table S4).

Table S2.

Mean 2-m air temperature (°C) and derived Pseudo-Lapse rates (°C/km) between stations of differing elevation (Fort Duchesne, elevation 1502 m; Five Mile, elevation 2280 m; Bruin Point, elevation 2973 m) for the months of April-September of each year from 2015-2020.

| **Year** | **Mean T (°C) at Ft. Duchesne** (low elevation) | **Mean T (°C) at Five Mile** (mid-slope) | **Mean T (°C)**  **at Bruin Point**  (high elevation) | **Average Pseudo-Lapse Rate (°C km^-1^)** (between low and mid elevation location) | **Average Pseudo-Lapse Rate (°C km^-1^)** (between mid and high elevation location) | **Average Pseudo-Lapse Rate (°C km^-1^)** (between low and high elevation location) |
| --- | --- | --- | --- | --- | --- | --- |
| **2015** | 18.26 | 15.54 | 10.20 | 3.49 | 7.71 | 5.48 |
| **2016** | 18.36 | 15.75 | 10.31 | 3.36 | 7.85 | 5.48 |
| **2017** | 18.82 | 15.68 | 10.50 | 4.04 | 7.46 | 5.66 |
| **2018** | 20.13 | 17.48 | 12.05 | 3.40 | 7.84 | 5.49 |
| **2019** | 18.20 | 15.43 | 10.13 | 3.58 | 7.66 | 5.51 |
| **2020** | 19.28 | 17.03 | 11.50 | 2.89 | 7.98 | 5.29 |

Table S3.

Mean wind speed (m s^-1^) for 3 stations of differing elevation in the Uinta Basin (Fort Duchesne, elevation 1502 m; Five Mile, elevation 2390 m; Bruin Point, elevation 2973 m) for the periods April – September 2015-2020.

| **Year** | **Mean Wind Speed (m s^-1^)**  **Ft. Duchesne**  (low elevation) | **Mean Wind Speed (m s^-1^)**  **Five Mile** (mid-slope) |
| --- | --- | --- |
| **2015** | 2.45 | 3.52 |
| **2016** | 2.58 | 3.92 |
| **2017** | 2.65 | 2.86 |
| **2018** | 2.57 | 4.12 |
| **2019** | 2.27 | 3.81 |
| **2020** | 2.46 | 4.01 |

Table S4.

Same as Table 3, except the lapse rates for only the afternoon hours of 13:00-16:00 MST . Mean Temperature (°C) and Pseudo-Lapse rate (°C/km) computed between stations of different elevation (Fort Duchesne, elevation 1502 m; Five Mile, elevation 2390 m; Bruin Point, elevation 2973 m) for the periods April – September 2015-2020.

| **Year** | **Average Pseudo-Lapse Rate (°C/km)** (between low and mid elevation location) | **Average Pseudo-Lapse Rate (°C/km)** (between low and high elevation location**)** |
| --- | --- | --- |
| **2015** | 8.23 | 7.46 |
| **2016** | 7.96 | 7.31 |
| **2017** | 8.82 | 7.53 |
| **2018** | 8.47 | 7.69 |
| **2019** | 8.60 | 7.45 |
| **2020** | 8.47 | 7.42 |

**References**

1. Horel, J. *et al.* Mesowest: cooperative mesonets in the western United States. *Bull. Am. Meteorol. Soc.* **83**, 211–225 (2002).

2. Mansfield, M. L. & Lyman, S. N. Winter Ozone Pollution in Utah’s Uinta Basin is Attenuating. *Atmosphere*  **12**, (2021).

3. Mansfield, M. L. & Hall, C. F. A survey of valleys and basins of the western United States for the capacity to produce winter ozone. *J. Air Waste Manage. Assoc.* **68**, 909–919 (2018).

4. Whiteman, C. D. & Hoch, S. W. Pseudovertical Temperature Profiles in a Broad Valley from Lines of Temperature Sensors on Sidewalls. *J. Appl. Meteorol. Climatol.* **53**, 2430–2437 (2014).
